# Joint Cache Content Placement and Task Offloading in C-RAN Enabled by Multi-Layer MEC

**DOI:** 10.3390/s18061826

**Published:** 2018-06-05

**Authors:** Haibo Mei, Kezhi Wang, Kun Yang

**Affiliations:** 1 School of Communication and Information Engineering, University of Electronic Science and Technology of China, Chengdu 611731, China; 2 Department of Computer and Information Sciences, Northumbria University, Newcastle upon Tyne NE2 1XE, UK; kezhi.wang@northumbria.ac.uk; 3 School of Computer Sciences and Electrical Engineering, University of Essex, Colchester CO4 3SQ, UK; kunyang@uestc.edu.cn or kunyang@essex.ac.uk

**Keywords:** cache content placement, user task offloading, Gale-Shaply method, population evolution game theory

## Abstract

In this paper, we work on a Cache and Multi-layer MEC enabled C-RAN (CMM-CRAN) to handle various user tasks with minimized latency and energy cost. We intend to solve two particular problems of CMM-CRAN. First, because CMM-CRAN has to maximally cache the most frequently requested data from Service Provide Server (SPS) to Remote Radio Head (RRH) and later offered to proximity mobile users, the cache content placement from SPSs to RRHs becomes a many-to-many matching problem with peer effects. Second, because of multi-layer MEC, a user task has to be dynamically controlled to be offloaded to the best fit cloud, i.e., either local MEC or remote MEC, to get served. This dynamic task offloading is a Multi-Dimension Multiple-Choice Knapsack (MMCK) problem. To solve these two problems, we provide a Joint Cache content placement and task Offloading Solution (JCOS) to CMM-CRAN that utilizes Proportional Fairness (PF) as the user scheduling policy. JCOS applies a Gale-Shaply (GS) method to work out the cache content placement, and a Population Evolution (PE) game theory coupled with a use of Analytic Hierarchy Process(AHP) to work out the dynamic user task offloading. According to the simulation results, CMM-CRAN with JCOS is proved to be able to provide highly desired low-latency communication and computation services with decreased energy cost to mobile users.

## 1. Introduction

Nowadays, large-scale field trials on C-RAN has been carried out in various provinces and cities across China. With long-term (around three years) commercial operation, the advantages of C-RAN have been demonstrated the network to be an effective Green Radio Network in terms of cost reduction, power saving, system performance improvement via interference mitigation and so on [1]. Further, with the increase in popularity of high definition video, gaming, virtual reality, more and more resource-hungry tasks come into play in User Equipment (UE) of C-RAN. It is then difficult for a UE to process those resource intense applications. This is because the resource of a UE, such as CPU, storage etc. is limited [2]. To solve this problem, MEC has been proposed to be incorporated into C-RAN to provide computing task offloading options to mobile users to extend their computation ability. In such MEC enable C-RAN, a UE can be lightly implemented and have extended battery life [3,4]. Therefore, one C-RAN, enabled by MEC, can work as a Green Radio Network to have low energy cost both on the network side and the user side.

In reality, C-RAN with MEC deployed in high level is still less able to provide highly desired low-latency computing and communication services to mobile users in low energy cost. This is because the fronthaul constraint is a major issue of C-RAN. In any C-RAN, the fronthaul capacity is normally constrained, so when a UE transmitting intense communication or computation task data to the Base Band Unit (BBU) or high level MEC through the constrained fronthaul, it may cause intolerable time delay and energy cost. Meanwhile, because the capacity of a frounthaul is limited, one fronthaul may not able to accommodate all the incoming UE requests. Also in C-RAN, the location-based social applications become more and more popular, and the social-aware traffic data over the fronthaul between RRH and the BBU pool surges with a lot of redundant information, which worsens the fronthaul constraint [5].

To solve the issue of constrained fronthaul, the idea of putting MEC to the lower edge of C-RAN becomes popular. In practice, lower edged cloud computing in C-RAN is able to locally handle UE computation tasks without going through fronthaul, then saves fronthaul cost. For example, ChinaMobile, with Huawei, ZTE and Nokia etc., has released the white paper on next generation C-RAN [4], which can dynamically split the functionalities of a BBU into a Centralized Unit and a Distributed Unit. A Distributed Unit could be deployed in lower edge and proximity of RRH to locally handle UE computing tasks for the sake of fronthaul cost saving. There have been numbers of work proposed to implement lower edged cloud into mobile networks. Fog computing-based RAN (F-RAN) [6,7] is one of the cutting edge network architectures proposed. In F-RAN, fog computing [8] extends MEC to reach a very low level, like device-to-device level, to more able to help the task offloading of proximity UEs. Unfortunately, F-RAN is still in its infancy. There are several outstanding problems that need further investigation, such as UEs transmission modes selection, interference suppression, UEs coordinated scheduling etc. [9]. Compared to fog computing, Multi-layer MEC is an easier and more practical way to put cloud computing to the lower edge of C-RAN, which has been comprehensively studied in [10]. In a multi-layer MEC enabled C-RAN, there is a High-level Edge Cloud (HEC) next to BBU to work as the central cloud to handle UE tasks. Meanwhile, there are numbers of Low-level Edge Clouds (LEC) individually close to RRHs to distributively handle proximity UE tasks. Multi-layer MEC follows the fact that MEC can be deployed in a distributed way throughout the network. In practice, because of the co-existence of HEC and LEC, Multi-layer MEC will lead to more complicated computation and communication resource allocation and a tricky procedure of cloud selection during user task offloading, i.e., dynamic task offloading, in C-RAN. There have been numbers of related work proposed to address similar challenges. For example, in [11,12,13], joint computation and communication resource allocation was studied for MEC enabled C-RAN. However, the work in [11,12,13] is not applicable to the multi-layer MEC scenario of C-RAN yet. In [14,15,16], the issue and solution on user dynamic task offloading were investigated in varieties of C-RANs supported by MEC. However, the existing work mainly tries to optimize user task offloading considering the energy or resource efficiency of the network, not the quality of service to mobile users. So far, there is no advanced work that can solve the user task offloading problem while guaranteeing low-latency service to mobile users yet. In this paper, multi-layer MEC and the solution on the dynamic task offloading problem are the focus of this paper.

To future release the fronthaul constraint in the face of redundant social-aware contents, cache is taken as another effective way to save fronthaul cost in C-RAN. In [17], cache was introduced into mobile networks, like heterogeneous small cell network, to relive the backhaul constraint and improve network performance. There also have been work trying to use cache to relive the backhaul constraint in MIMO Interference Networks [18], and content-centric wireless network [19]. There are also numbers of work done to solve the issues on cache working with various of networks, like cache content placement, cache content update and deliver etc. [20]. For example in [21], the author modeled the cache content placement issue in small base stations as a many-to-many matching problem in wireless small cell networks. Similar to the work in [17,18,19], if cache working in C-RAN, part of the frequent requested data of a UE could be placed in cache area of proximity RRH, and delivered to the requesting UE without going through fronthaul. This will greatly decrease the latency of a each user task. According to our best knowledge, few works so far apply caches to help the performance of C-RAN, and there is no work applying caches to co-work with multi-layer MEC to cooperatively improve the performance of C-RAN.

In this paper, we focus on the C-RAN that works with a setting of multi-layer MEC and advanced Maximum Distance Separable (MDS)-code-based cache. The main work of this paper is to solve two particular problems of CMM-CRAN. First, because CMM-CRAN has to maximally cache the most frequently requested data locally in RRH, the cache content placement from SPSs to RRHs is a many-to-many matching problem with peer effects. Secondly, because of multi-layer MEC, a user task in CMM-CRAN has to be controlled to be dynamically offloaded to the bet fit cloud, i.e., either to LEC or HEC. We define such user task offloading problem as a MMCK problem. The main work and primary contributions of this paper are:

1. We design a Joint Cache content placement and task Offloading Solution, named JCOS, to solve those two problems of CMM-CRAN. With JCOS, UE tasks in CMM-CRAN are easier to obtain the frequently requested content through cache, and the computation tasks can be handled by the best fit edge cloud guaranteeing the benefits of both mobile users and the network. Therefore, JCOS could effectively save UE task latency, energy cost and fronthaul capacity, then improve the performance of CMM-CRAN.
2. JCOS utilizes the well known GS method to come up a Cache Content Placement Algorithm(CCPA) to solve the many-to-many matching problem on cache placement. CCPA considers the storage capacity of each RRH, the fronthaul and RF link capacities, and the content popularity to solve the matching problem.
3. JCOS also applies the PE game theory coupled with a use of a AHP as the method to solve the MMCK problem on user task offloading. The PE method works out the offloading choices based on a series of comparisons of cloud selection utilities. A cloud selection utility is associated to cloud capacity constraint, fronthaul constraint, and RF constraint.
4. The CCPA on cache and PE method on dynamic task offloading work jointly in JCOS to have acceptable complexity, stability and salability.

The remainder of this paper is organized as follows. In Section 2, we describe CMM-CRAN system model and formulate the problems on cache content placement and user task offloading. In Section 3, we present JCOS to solve the problems. In Section 4, simulation results and analysis are presented, where we discuss and analyze the pros and cons of JCOS. In Section 5, we give conclusions and future work.

## 2. Model and Problem Formulation

### 2.1. CMM-CRAN Model

**Figure 1:**
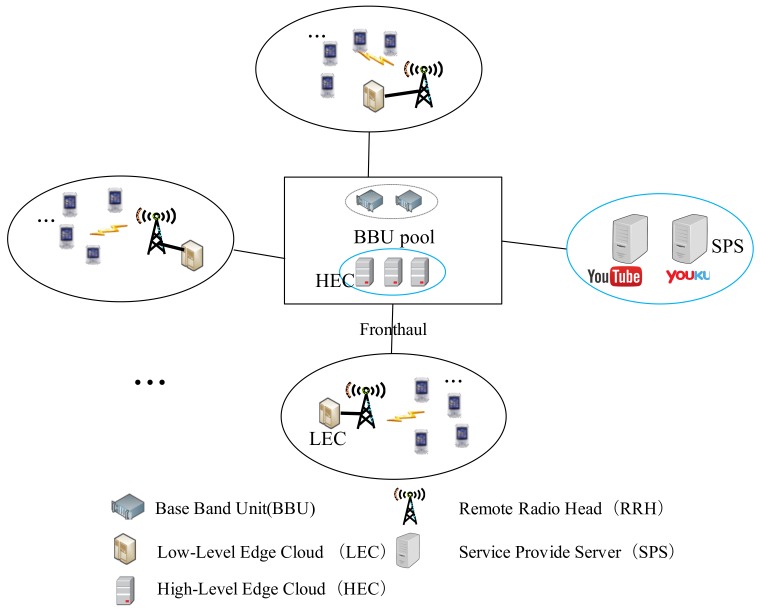
System Architecture of CMM-CRAN.

In CMM-CRAN, the multi-layer MEC is shown in Figure 1, where the LEC near a RRH is mainly used to locally serve the tasks of proximity UEs to save fronthaul cost, while the HEC next to the BBU pool is mainly deployed to remotely serve the tasks offloaded from UEs. From users’ perspectives, whether a UE will be served by a LEC or HEC is a user task offloading problem i.e., a MMCK problem. To solve the problem, one solution not only has to consider the latency and energy cost requirement of UE tasks, but also the cloud capacity constraint, fronthaul constraint, and RF constraint.

**Figure 2:**
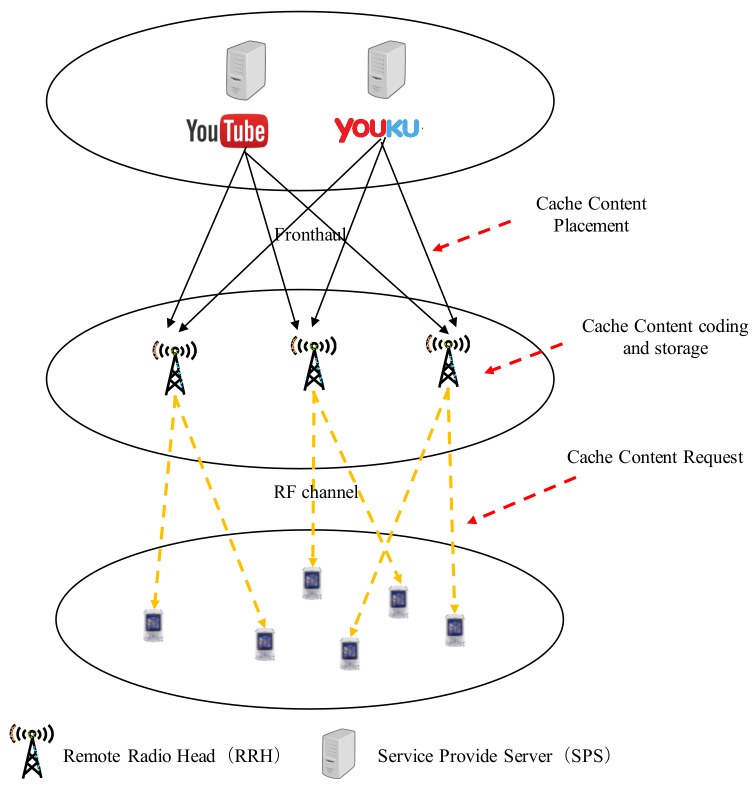
The many-to-many matching problem from SPSs to RRHs in CMM-CRAN.

On the other hand, in Figure 1 there are numbers of SPSs to provide social-aware contents, like viral videos from popular video websites (YouTube, Netflix, Youku, etc.), which are frequently shared, quoted and downloaded by mobile users. To save fronthaul cost, it will be great helpful to cache those coded social-aware contents in each RRH of CMM-CRAN, where UEs can obtain most part of the coded social-aware contents directly from their proximity RRH instead of from SPSs through fronthaul. In practice, it requires a cache content placement mechanism to decide which coded social-aware content to be placed to which RRH to maximally save the fronthaul cost. Such cache content placement, as shown in Figure 2, is a many-to-many matching problem from SPSs to RRHs with peer effects, and related solution should make sure that a cached content must serve as many user requests as possible in proximity of each RRH. To do so, a solution has to take the factors, including storage capacity of each RRH, the fronthaul capacity, and the content popularity, into considerations to solve the matching problem.

### 2.2. Problem Formulation

#### 2.2.1. UE Task, Latency and Energy Cost

In C-RAN enabled by MEC, we assume that a UE *i* in the set I={1,2,3,…,I} has task $U_{i}$ as follow

(1) $$\begin{array}{c} U_{i}=\left(F_{i},D_{i}\right),\;\;\forall i\in I \end{array}$$

where $F_{i}$ describes the total number of the CPU cycles requested by task $U_{i}$. $D_{i}$ denotes the size of the data transmitted from RRH to UE *i* through RF channel. $D_{i}$ also is the size of the data transmitted from cloud to UE *i* through fronthaul after task execution, including task’s output parameter and calculation results etc. [11].

Based on the UE task defined, the latency of finishing task $U_{i}$ is formulated as

(2) $$\begin{array}{c} T_{i}=\frac{F_{i}}{f_{ij}}+\frac{D_{i}}{r_{ij}}+\frac{D_{i}-D_{i}^{c}}{r_{j}^{F}} \end{array}$$

where a RRH *j* is the radio access point in the set J={1,2,3,…,J}. $f_{ij}$ is the computation capabilities allocated from cloud to serve UE *i* through RRH *j*. $r_{ij}$ is the data rate of the RF channel serving UE *i*. $r_{j}^{F}$ is the data rate of fronthaul connecting RRH *j*. $D_{i}^{c}$ is the size of data provided by cache. Compared to standard C-RAN, in the cache enabled C-RAN, only the part of data with a size of ($D_{i}-D_{i}^{c}$) of task $U_{i}$ needs to be offloaded to cloud through fronthaul, instead of all the data $D_{i}$. According to (2), the latency of a UE task is cumulatively caused by the cloud computing, RF transmission, and data transmission in fronthaul. The latency of a task is closely related to the computation and communication resource allocated to the task.

We define $E_{i}$ as the energy cost of the UE task $U_{i}$, which is formulated as

(3) $$\begin{array}{c} E_{i}=\varphi {\left(f_{ij}\right)}^{\vartheta -1}F_{i}+\eta P_{j}\left(\frac{D_{i}}{r_{ij}}\right) \end{array}$$

where RRH *j* is the radio access point, φ is the effective switched capacitance and ϑ≥1 is the positive constant [11]. According to the realistic measurements, φ can be set to $\varphi =10^{-11}$. η≥0 is a weight to the tradeoff between the energy consumptions in the mobile cloud and C-RAN, and it can be also explained as the inefficiency coefficient of the power amplifier at RRH. $P_{j}$ is the power of RRH *j*. The energy cost of a task is directly related to the computation and communication resource allocated to the task. According to (3), the energy cost of a UE task is cumulatively caused by the cloud computing and RF transmission. For the simplicity of this paper, we ignore the energy cost of fronthaul in this paper, as the energy cost issue in fronthaul is not critical to the performance of the network.

In C-RAN, we define a Computing Block (CB) as the atom computation resource unit, which has 1 CPU cycle as the computation capacity. Accordingly, the computation resource allocated to UE task $U_{i}$ in RRH *j* is represented as

(4) $$f_{ij}=\sum_{f=1}^{F_{j,max}}\beta_{ijf}$$

where $\beta_{ijf}$ denotes whether CB *f* allocated to task $U_{i}$ ($\beta_{ijf}=1$) or not ($\beta_{ijf}=0$) in RRH *j*. $F_{j,max}$ represents the number of CBs available to task $U_{i}$ in RRH *j*, which is provided by the MEC. The CB allocation follows a form of proportional fairness policy [22] in this paper.

The UE data rate in RF link of C-RAN is close related to the outputs of the communication resource allocation and scheduling method employed. The outputs affect the quality of the RF links. Similarly to computation resource, in this paper, the Orthogonal Frequency Division Multiplexing (OFDM) based communication resource is represented as fixed number of atom Radio Blocks (RBs) grouped as *K*, with total bandwidth *B*. We employ Signal to Interference plus Noise Ratio (SINR) to evaluate the channel quality of each RB. Specifically, if a UE *i* associated to RRH *j* with RB *k* allocated, its channel gain $h_{ijk}$ is formulated as

(5) $$h_{ijk}=G_{ij}^{0}-\left(20\cdot \log\left(\frac{4\pi d_{0}}{\psi }\right)+10\cdot \gamma \cdot \log\left(d_{ij}/d_{0}\right)+X_{\varrho }\right)$$

where $G_{ij}^{0}$ is the antenna gain between UE *i* and RRH *j*, the distance between UE *i* and RRH *j* is $d_{ij}$. According to the channel gain model in [23], $d_{0}$ = 100 m, and $d_{ij}>d_{0}$. γ is the path-loss exponent, which is a constant. ψ is the wave length in meters. $X_{\varrho }$ describes the random shadowing effects, and follows the normal distribution with zero mean and $\varrho^{2}$ variance, i.e., $X_{\varrho }∼N\left(0,\varrho^{2}\right)$. Based on the channel gain defined in (5), SINR of RB *k*: $S_{ijk}$ is formulated as

(6) $$S_{ijk}=\frac{\frac{P_{j}}{K}\cdot h_{ijk}}{\sum_{\forall t\in Q_{j}}\frac{P_{t}}{K}\cdot h_{itk}+N_{0}}$$

where $P_{j}$ is the power of RRH *j*. $N_{0}$ denotes the estimated power of noise under the cell coverage of RRH *j* (in dBm). $Q_{j}$ is the group including all the external and proximity interfering RRHs to RRH *j*. A UE will receive inter cell interference from the RRHs in group $Q_{j}$, if its allocated RBs are used by those interfering RRHs in $Q_{j}$ at the same time.

According to the RB SINR formulated in (6), the data rate of RB *k* serving UE *i* in RRH *j* can be expressed as

(7) $$\begin{array}{c} r_{ijk}=B\cdot {log}_{2}\left(1+S_{ijk}\right) \end{array}$$

The data rate of UE *i* served by RRH *j* is formulated as

(8) $$\begin{array}{c} r_{ij}=\sum_{k=1}^{K}\alpha_{ijk}\cdot r_{ijk} \end{array}$$

where $\alpha_{ijk}$ represents the RB allocation policy for UEs in RRH *j*. $\alpha_{ijk}=1$ means RB *k* is allocated to UE *i*, while $\alpha_{ijk}=0$ means not. Similar to CB allocation, the RB allocation also follows the proportional fairness policy in this paper.

#### 2.2.2. Formulate the Cache and Task Offloading Problems

In CMM-CRAN, UEs in set I get computation and communication services through *J* RRHs in the set J={1,2,3,…,J}. A UE intends to associate to its closest RRH for the best RF link. We define $R_{j}$ as the set of all the UEs associated to RRH *j*, and $I={⋂}_{j=1}^{J}R_{j}$. As discussed, the service providers can cache their social-aware content in the RRHs such that each RRH *j* can locally serve a UE *i* via a radio link. We define, in set J, each RRH *j* has a cache storage constraint $q_{j}$.

We suppose that the *I* UEs try to obtain data chosen from a library of *V* contents in the set V={1,2,3,…,V} provided by SPSs. According to the features of social network, each UE *i* in set I has interest $t_{iv}$ to content *v* in the set V, which can be calculated by the method proposed in [21]. We parametrize MDS codes by $\left(l_{v},n_{v}\right)$ such that content *v* is cut into $n_{v}$ fragments each in a constant size *s*, and then coded into $l_{v}$ independent packets by MDS. Any $n_{v}$ packets can rebuild the entire content [20]. Considering that the RRH *j* caches $m_{jv}$ coded packs of content *v*, we have $M^{j}=\left[m_{j1},m_{j2},m_{j3},\ldots ,m_{jV}\right]$ as the cache content placement vector of RRH *j* considering all the contents in the set V.

In this paper, the cache content placement is modeled as a many-to-many matching game, where the set V of contents and the set J of RRHs are two teams of players. The matching is defined as an assignment of contents in V to RRHs in J. A RRH *j* stores contents depending on its storage capacity $q_{j}$ and the interests of its UEs to those contents i.e., $t_{iv}\;\left(i\in R_{j},v\in V\right)$. In addition, a SPS prefers caching content to the RRH which downloads data in a quicker speed. We define the matching problem in this paper as

**Definition** **1.** *The many-to-many matching*μ* for the cache content placement problem is a mapping from the set*V∪J*into the set of all subsets of*V∪J*such that for every*v∈V*and*j∈J:

*1.*  μ(v)*is contained in*J*and*μ(j)*is contained in*V;
*2.*  |μ(v)|≤J*for all v in*V;
*3.*  $|\mu \left(j\right)\cdot M^{j}\leq q_{j}|$*for all j in*J;
*4.*  *j is in*μ(v)*if and only if v is in*μ(j);

*with*μ(v)*being the set of player v ’s partners under the matching*μ.

In Definition 1, the mangy-to-many matching μ follows conditions (1)–(4). Condition (2) denotes that each content *v* can maximally be cached to all the *J* RRHs in the set of J. In addition, condition (3) denotes each RRH *j*, caching fragments of all the contents, is under its storage constraint of $q_{j}$.

In CMM-CRAN, the matching of contents to RRHs should be done consider the constraint formulated as

(9) $$\begin{array}{c} C1:\left(\sum_{\forall v\in V}\sum_{\forall i\in R_{j}}t_{iv}\times m_{jv}\right)\geq I_{min},\;\;\left(\forall j\in J\right) \end{array}$$

where C1 denotes that users’ most interested social-aware contents should be maximally cached in RRHs to save fronthaul cost in CMM-CRAN. In (9), the overall interests of UEs to any content *v* in RRH *j* should be higher than the pre-configured overall interests $I_{min}$.

In addition, the matching also needs to consider two constraints formulated as

(10) $$\begin{array}{c} C2:q_{j}\geq \sum_{\forall v\in V}m_{jv},\;\left(\forall j\in J\right) \end{array}$$

(11) $$\begin{array}{c} C3:m_{jv}\leq n_{v},\;\left(\forall v\in V,\;\;\forall j\in J\right) \end{array}$$

where C2 denotes the overall size of the cached contents in each RRH *j* should not exceed the storage constraint $q_{j}$. C3 constraints the number of fragments of a content *v* not to exceed $n_{v}$ when cached in RRH *j*. This is to save the storage capacity of RRH *j* while satisfying the minimal requirement of re-constructing the content *v* in RRH *j*.

In CMM-CRAN, because of multi-layer MEC, a UE *i*, associated to RRH *j*, has two task offloading options i.e., either to the LEC or the HEC. Thus, one has

(12) $$C4:a_{ij}=\left\{0,1\right\},\left(\forall i\in R_{j},\forall j\in J\right)$$

where $a_{ij}=1$ represents UE task $U_{i}$ offloaded to LEC, and $a_{ij}=0$ represents UE task $U_{i}$ offloaded to HEC in the coverage of RRH *j*. Also, one has

(13) $$C5:\sum_{\forall j\in R_{j}}a_{ij}\leq 1,\;\left(\forall j\in J\right)$$

which denotes each UE task can only be executed in one cloud through a RRH. A solution to the user task offloading problem is to make sure each UE task being controlled to be offloaded to the best fit cloud to save task latency.

Considering a UE task $U_{i}$, which downloads content *v* in the set V through RRH *j*, the latency and energy cost of $U_{i}$ is re-formulated out of (2) and (3) as

(14) $$T_{i}=\frac{F_{i}}{f_{ij}}+\frac{n_{v}\cdot s}{r_{ij}}+\left(1-a_{ij}\right)\times \frac{(n_{v}-m_{jv})\cdot s}{r_{j}^{F}},\left(\forall i\in R_{j}\right)$$

(15) $$\begin{array}{c} E_{i}=\varphi {\left(f_{ij}\right)}^{\vartheta -1}F_{i}+\eta P_{j}\frac{n_{v}\cdot s}{r_{ij}},\left(\forall i\in R_{j}\right) \end{array}$$

where $n_{v}$ is the number of coded data packs required to re-construct content *v*. Each coded pack is in a size of *s*. According to (14), if UE *i* offloading task to LEC, i.e., $a_{ij}=1$, there will be no latency caused by fronthaul transmission.

To formulate delivering low-latency and low energy cost services in CMM-CRAN, one has

(16) $$C6:T_{i}\leq T_{i,max},\;\left(\forall i\in R_{j},\;\forall j\in J\right)$$

(17) $$C7:E_{i}\leq E_{i,max},\;\left(\forall i\in R_{j},\;\forall j\in J\right)$$

where $T_{i,max}$ and $E_{i,max}$ are the maximal allowed latency and energy cost of UE task $U_{i}$.

Moreover, for the mobile edge cloud, it cannot have unlimited computation capacity, or unlimited computation power. Therefore, for cloud, one has

(18) $$C8:\sum_{\forall i\in R_{j}}f_{ij}\leq F_{j,max},\;\left(\forall j\in J\right)$$

where $F_{j,max}$ denotes the maximum computational capacity available in RRH *j*. As discussed, $F_{j,max}$ is composed by the computation capacity of the LEC in proximity of RRH *j* and the part of the computation capacity from the HEC.

The optimization problem therefore is formulated as

(19) $$\begin{array}{cc} P:\;\;\; & \min_{m_{jv},a_{ij}}\;\;\;\left(\sum_{\forall i\in R_{j}}T_{i}\right),\;\left(\forall j\in J\right)\\ & \mathrm{subject}\;\mathrm{to}\;:\;C1-C8\; \end{array}$$

where P denotes minimizing the overall task latency in each RRH, following the many-to-many matching output on cache and user task offloading strategy, i.e., $\left(m_{jv},a_{ij}\right)\;\left(\forall i\in R_{j},\;\forall j\in J,\;\forall v\in V\right)$. P is subjected to the constraints of C1−C8. Since problem P considers minimizing overall task latency with energy cost constrained by C7, the solution of problem P thus can make sure CMM-CRAN delivering low-latency services with low energy cost.

## 3. Solutions

To solve the problem P, we first design a Cache Content Placement Algorithm(CCPA)-based on GS method to solve the many-to-many matching problem of cache content placement. Second, we discuss how to use PE coupled with the use of AHP to solve theMMCKproblem on user task offloading. Finally, we provide JCOS to get the optimal cache content placement and user task offloading outputs by jointly carrying out the procedures of CCPA and PE.

### 3.1. Cache Content Placement Algorithm

CCPA employs the well known GS method to realize optimal cache content placement. The GS method is also named as deferred-acceptance method, and was proposed by *D*. Gale and L. Shapley in 1962 to work out the problems of college admission and marriage stability [24]. GS method is further applied to find stable matching to different problems, like job matching [25], etc. In this paper, CCPA assigns cache contents to RRHs to obtain $m_{jv}\;\left(\forall j\in J,\;\forall v\in V\right)$ as the outputs of a stable match, which is under the constraint of C1−C3. To do so, contents and RRHs as players each needs to specify its preferences over subsets of the opposite set based on its goal in the network.

#### 3.1.1. Preferences of RRHs and Contents

The preference of RRH *j* to content *v* is formulated as

(20) $$I_{jv}=\sum_{\forall i\in R_{j}}t_{iv}$$

where $I_{jv}$ denotes that the interest of RRH *j* to content *v* is the sum of the interests of all the associated UEs of RRH *j* to content *v*.

Considering the interest of a content *v* to a RRH *j*, content *v* would be preferred to be cached at the RRH *j* that offers the shortest download time for the expected requesting UEs. When a UE task $U_{i}$ offloaded to HEC in RRH *j* (i.e., $a_{ij}=0$), the download time depends on the capacity of the fronthaul link $r_{j}^{F}$ and the RF link $r_{ij}$ that connects the RRH *j* to the UE *i*. The content is first downloaded by RRH *j* which then serves the UEs. Thus, in the worst case, downloading a content *v* takes the required time to pass by the link with the poorest capacity. On the other hand, when a UE task $U_{i}$ offloaded to LEC in RRH *j* (i.e., $a_{ij}=1$), the download time only depends on the RF link $r_{ij}$. Therefore, when many UEs are expected to request the same content from RRH *j*, the download time is given by:

(21) $$T_{Dj}=\frac{\sum_{\forall i\in R_{j}}\left(\left(1-a_{ij}\right)\times \frac{1}{r_{j}^{F}}+\frac{1}{r_{ij}}\right)}{|R_{j}|}$$

We use the notation $G_{1}{≻}_{v}G_{2}$ to imply that content *v* prefers to be stored in the RRHs set $G_{1}\left(G_{1}\subseteq J\right)$ than stored in the ones proposed in $G_{2}\left(G_{2}\subseteq J\right)$, according to the content downloading time formulated in (21). A similar notation is used for the RRHs to set a preference list for each content, according to the RRH preferences over contents formulated in (20). Faced with a set *G* of possible partners, a player *v* can determine which subset of *G* it wishes to match to. We denote this choice set, i.e., preference list, by $P_{v}\left(G\right)$.

#### 3.1.2. Algorithm Design

In this paper, we are interested to look at a stable solution of the many-to-many matching μ within RRHs and contents. In the stable matching, there will be no players that are not matched to one another but they all prefer to be partners. We design CCPA to reach the pairwise stability [21] following the preferences of contents and RRHs as defined in (20) and (21). CCPA is defined as Algorithm 1. According to [21], Algorithm 1 will surely converge to the pairwise stability within RRHs and contents and get the optimal outputs $M^{j}\;\left(\forall j\in J\right)$ as the results.

In Algorithm 1, there are four phases. During the first phase, SPSs and RRHs collect the required parameters, such as the fronthaul capacity, RF link capacity, RRH cloud capacity, and users’ interests to contents, to define the preferences. Then the preferences of RRH to content, i.e., $I_{jv}$, and RRH content downloading time, i.e., $T_{Dj}$, are calculated. In the second phase, based on $\left(I_{jv},T_{Dj}\right)$ (∀j∈J,∀v∈V), SPSs initiatively define the preference list for each owned content over the set of RRHs as: $P_{v}\left(J,0\right)\;\left(\forall v\in V\right)$. In the mean time, the RRHs initiatively define their preferences list over the set of contents that would be proposed by the SPSs as: $P_{j}\left(V,0\right)\;\left(\forall j\in J\right)$. Afterwards, Algorithm 1 goes into the third phase to work out the matching through a finite repetition. Specifically, in the *t*-th repetition, each content *v* is proposed to its current most preferred set of RRHs: $P_{v}\left(J,t\right)$. Then, each RRH *j* rejects all the contents but its most interested ones from the set of alternatives proposed to itself. Each RRH *j* carries out the rejection according to its preference list: $P_{j}\left(V,0\right)$, and any proposed contents not in the list will be rejected. After those rejections, each content *v* then updates its preference list to $P_{v}\left(J,\left(t+1\right)\right)$ that includes the RRHs to which it previously proposed to and have not rejected itself yet. Obviously, we have $P_{v}\left(J,\left(t+1\right)\right){≻}_{v}P_{v}\left(J,t\right)$. Then, the matching goes into next (t+1)-th repetition. The matching repetition will keep on running until no rejection is issued i.e., Re=No, which means the matching converges into a stabilized pairwise state. Based on the stable matching, in the last phrase, Algorithm 1 carries out the cache content placement according to the matching output: $P_{v}\left(J,t\right)\;\left(\forall v\in V\right)$, where any $P_{k}\left(J,t\right)$ denotes the finalized stable matching of content *k* to the RRHs in the set of J. During the content placement, the fragments $n_{k}$ of content *k* will be cached to each RRH *j* in the set of $P_{k}\left(J,t\right)$, i.e., $m_{jk}=n_{k}$. The cache content placement follows the capacity constraint of each RRH, and if the real time capacity $a_{j}$ of RRH *j* exceeds constraint $q_{j}$, RRH *j* will not receive content any more. Finally, $M^{j}$ (∀j∈J) is worked out as the cache content placement results.
**Algorithm 1:** Cache Content Placement Algorithm.
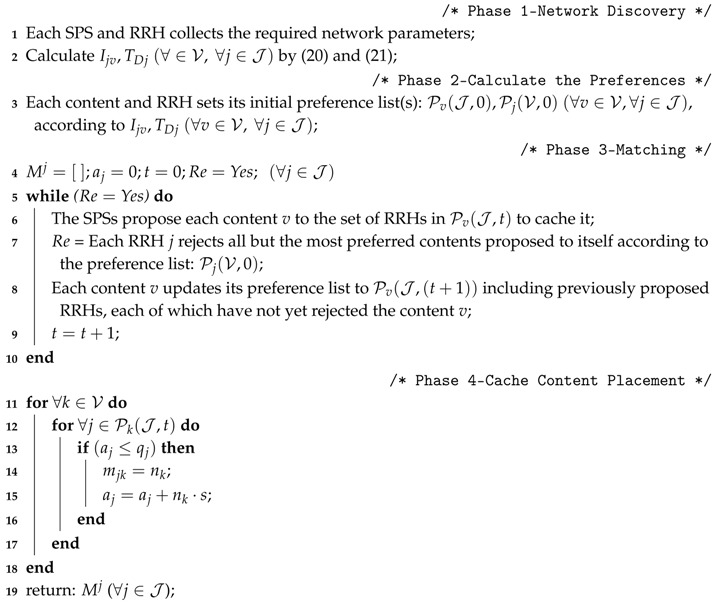


### 3.2. PE Method on User Task Offloading

As discussed, the user task offloading problem is a MMCK problem, and a UE task $U_{i}$ under the coverage of a RRH can be either offloaded to the HEC or the LEC. Thus, UE *i* has to be controlled to carry out a way of cloud selection, which takes numbers of dependent elements into consideration. In practice, UE *i* should select the cloud to save the offloading time and energy cost, while not violating the fronthaul constraint, the cloud capacity constraint, the RF constraint, nor causing load un-balance of each candidate cloud. However, it is a challenge to fully satisfy all the requirements. This is because it has to address the situation that different UE tasks are in the face of different cache support, latency constraints, energy constraints and data volumes. Also the situation of CMM-CRAN, in terms of cloud capacities, UEs distributions and RB interference, is changing all the time. Therefore, user task offloading is a complicated problem. In this paper, we apply a game theory, named PE [26], to solve the complicated user task offloading problem.

#### 3.2.1. Population Evolution Game

Game theory has been comprehensively surveyed in [27]. It is also surveyed in [28] on how to work into communication networks. However, there is not much work so far that applies game theory on MEC enabled C-RAN yet. Some works in [29,30] have used game theory to solve network routing and wireless network selecting problems. In this paper, PE, as a type of evolution game theory [31], is applied to solve the user task offloading problem. PE basically simulates population growth of speeches, like fishes in a swamp, in real world. It follows the principle that if there are more resource (higher utility), the population will grow (more individuals joining), verse visa. This principle is formulated as

(22) $$\begin{array}{c} {\dot{x}}_{k}^{g}=\sigma \times x_{k}^{g}\times \left(U_{k}^{g}-{\bar{U}}_{g}\right) \end{array}$$

where in each period, the individuals observe the utility of choosing strategy *k*, i.e., $x_{k}^{g}$, and the utility and average utility of the entire population *g*, i.e., $U_{k}^{g}$ and ${\bar{U}}_{g}$. In next step, the utility of choosing strategy *k* is adapted to ${\dot{x}}_{k}^{g}$ accordingly. The higher utility of this strategy, the more individuals will choose it in the group. A strategy could be leaving this group, staying in the group or newly joining the group. The adaptation is carried out with the help of replicator dynamics, where σ is the gain for the rate of strategy adaptation. PE is normally used on decision making based on utility comparisons.

PE is applicable to our cloud selection problem, because we can take HEC or LEC as blocks of computation resource. A cloud, like a fish swamp to fishes, can handle numbers of UEs’ tasks. If a cloud is more suitable for UEs, it can have high cloud selection utility to persuade more UEs joining this cloud. This is very similar to the fish swamp example, and obviously can be modeled by (22). Compared to classic games, like Nash equilibrium, Stackelberbg equilibrium [27,28], PE could converge to the global equilibrium in a easier procedure, and it not only guarantees the benefits of the individual game players but also the benefits of the populations formed by players. Therefore PE is more applicable to the user task offloading problem of this paper.

#### 3.2.2. Calculate Cloud Selection Utility by AHP

In this paper, the cloud selection utility is calculated by AHP and further utilized in PE to work out the user task offloading. AHP has been widely used on complicated decision-makings in different fields such as government, business, industry, healthcare, and education [32]. As known in real work, it is complicated and difficult to make correct decisions considering large numbers of dependent elements. The elements can relate to any aspect of the decision problem, and could be tangible or intangible, carefully measured or roughly estimated, well or poorly understood that applies to the decision at hand. Each element has different influence to the final decision. Therefore, there is no absolute wrong or correct decision, and the AHP helps decision makers to find one that best suits their goal and their understanding of the problem. In practice, whether a decision is suitable or not to the problem is reflected by its utility considering related elements.

To the user task offloading problem of this paper, AHP calculates the cloud selection utilities considering the cache, cloud capacity, the fronthaul constraint, and the RF constraint. Specifically, the utilities of UE *i* choosing cloud HEC and LEC in RRH *j* are formulated as $U_{ij}^{H}$ in (23) and $U_{ij}^{L}$ in (24).

(23) $$\begin{array}{c} U_{ij}^{H}=\omega_{i}^{1}\cdot \frac{f_{H}}{f_{max}^{H}}+\left(\omega_{i}^{2}\cdot \frac{r_{j}^{F}}{r_{max}^{F}}+\omega_{i}^{3}\cdot \frac{r_{ij}}{r_{j,max}}\right)\cdot \left(1+\frac{m_{jv}}{n_{v}}\right) \end{array}$$

In (23), $f_{H}$ and $f_{max}^{H}$ are the real time and maximal computation capacities of HEC. $r_{j}^{F}$ and $r_{max}^{F}$ are the real time and maximal data rate of the fronthaul connecting HEC and RRH *j*, which is closely related to fronthaul capacity constraint. $r_{ij}$ and $r_{j,max}$ are the real time and maximal data rate of the RF link connecting UE *i* to RRH *j*, which is closely related to RF constraint in RRH *j*. Basically, (23) denotes that if the HEC, the fronthaul and the RF link have rich capacities in a RRH, the utility of selecting the HEC will be high, vice versa.

(24) $$\begin{array}{c} U_{ij}^{L}=\omega_{i}^{1}\cdot \frac{f_{L}}{f_{j,max}^{L}}+\left(\omega_{i}^{2}\cdot 1+\omega_{i}^{3}\cdot \frac{r_{ij}}{r_{j,max}}\right)\cdot \left(1+\frac{m_{jv}}{n_{v}}\right) \end{array}$$

In (24), $f_{L}$ and $f_{j,max}^{L}$ are the real time and maximal computation capacities of LEC. Because LEC is locally deployed in proximity of RRH *j*, UE *i* does not need to consider the fronthaul capacity as the AHP element in (24). Thus, compared to the utility $U_{ij}^{H}$ of HEC, the value to the element of fronthaul data rate in (24) is always 1. Generally, (24) denotes that if the LEC and the RF link have rich capacities in a RRH, the utility of selecting the LEC will be high, vice versa. Also because no need to consider fronthaul constraint, LEC is highly possible to be more attractive to UE tasks compared to the HEC. In addition, both in (23) and (24), the cloud selection utility $U_{ij}^{H}$ and $U_{ij}^{L}$ are close related to the cache content placement. Basically, if more content of the objective content *v* being cached in RRH *j*, i.e., $\frac{m_{jv}}{n_{v}}$ in higher value, the utility could be higher, vice versa.

In (23) and (24), $\omega_{i}^{1}$, $\omega_{i}^{2}$ and $\omega_{i}^{3}$ are the weights determining how the element of cloud capacity, fronthaul constraint, and RF constraint effect the utility respectively. $\omega_{i}^{1}$, $\omega_{i}^{2}$ and $\omega_{i}^{3}$ are calculated through (27). To calculate each weight, we should firstly estimate the sensitivenesses of each UE to each element, which are measured by integer values between 1 and 9 [33]. Table 1 lists the sensitivity measurements as examples. In Table 1, the higher the value is, more sensitive the UE to the element is. For example, when selecting a cloud for task offloading, a UE with voice task will be highly sensitive to fronthaul and RF constraints (8, 9 as the value), while not sensitive to cloud capacity (2 as the value). A UE with data-process task, like artificial intelligence computing, will be highly sensitive to cloud capacity (8 as the value) and lowly sensitive to fronthaul and RF constraints (1 and 3 as the value). A UE with data-stream task, like content downloading, will be medially sensitive to cloud capacity, fronthaul and RF constraints. Particularly, if a UE with multi-media task, like online gaming, it will be almost highly sensitive to all the elements. Based on those sensitiveness measurements, we secondly carry out a series of pairwise comparisons between all pairs of AHP elements to evaluate the relative sensitiveness of one element over another, considering specific type of UE task. For example, considering data-process type of UE task, the pairwise comparisons of the sensitiveness data in Table 1 are shown in (25). (25) results a 3×3 square matrix *c*, where $c_{ij}$ denotes the pair comparison between element *i* and *j*.

(25) $$\begin{array}{c} c=\left(\begin{array}{ccc} c_{11} & c_{12} & c_{13}\\ c_{21} & c_{22} & c_{23}\\ c_{31} & c_{32} & c_{33} \end{array}\right)=\begin{array}{c} \left(\begin{array}{ccc} 8/8 & 8/1 & 8/3\\ 1/8 & 1/1 & 1/3\\ 3/8 & 3/1 & 3/3 \end{array}\right) \end{array} \end{array}$$

Based on (25), for a given type of UE task, the eigenvector for each element, say element *k*, can be calculated using the geometric mean method given in (26).

(26) $$\begin{array}{c} e_{k}=\sqrt[{3}]{c_{k1}\times c_{k2}\times c_{k3}},\;k=1,2,3 \end{array}$$

In (27), the normalization of $e_{k}$ will determine the weight: $\omega_{i}^{k}$ of element *k* to the UE *i* with the given type of task.

(27) $$\begin{array}{c} \omega_{i}^{k}=\frac{e_{k}}{\sum_{k=1}^{3}e_{k}},\;\;k=1,2,3 \end{array}$$

### 3.3. Joint Solution on Cache Content Placement and User Task Offloading

JCOS in this paper is to realize effective user task offloading and jointly work out the cache content placement in CMM-CRAN. To accomplish a joint solution to the cache content placement and user task offloading problems, JCOS intends to solve problem P by applying CCPA and PE method to form a single algorithm. This is plausible, as the cache and multi-layer MEC are closely co-related in CMM-CRAN. For example, according to CCPA, the preferences of contents over the RRHs, as defined in (21), are close related to the result of user task offloading: $a_{ij}$ ($\forall j\in J,\;\forall i\in R_{j}$). Another example is that the cloud selection utilities, as defined in (23) and (24), are close related to the output of cache content placement: $m_{jv}$ (∀j∈J,∀v∈V). These provide a strong basis that JCOS can jointly solve the cache and user task offloading problems.

JCOS is demonstrated as Algorithm 2, which iteratively carries out the cache content placement and cloud selection for all the UEs in CMM-CRAN, while each UE has a specific type of task, e.g., voice call, data-process, data-stream and multi-media, at a time. Consider the UEs in the set $R_{j}$ of RRH *j*, we define that the part of UEs selecting the HEC form a HUE population: $H_{j}$, and the part of UEs selecting the LEC form a LUE population: $L_{j}$, and $R_{j}=H_{j}\cap L_{j}$.

In the beginning, Algorithm 2 carries out a Random Access (RA) method to obtain the cache content placement as $m_{jv}\;\left(\forall v\in V\right)$ at step 1. The RA method means each content is randomly cached to RRHs. Also, Algorithm 2 assumes that each UE in the set $R_{j}$ of RRH *j* initially selects the cloud that offers itself the highest utility (step 4–11), which is a greedy approach. Then, Algorithm 2 updates the utilities of UEs selecting clouds (step 12). Algorithm 2 also calculates the average utility: ${\bar{U}}_{j}^{H}$ and homogeneous utility: ${\overset{ˇ}{U}}_{j}^{H}$ of the newly formed HUE population, and the average utility: ${\bar{U}}_{j}^{L}$ and homogeneous utility: ${\overset{ˇ}{U}}_{j}^{L}$ of the new LUE population (step 13–14). The homogeneous utility of a UE population represents the utility i.e., payoff of the entire population. According to PE, if a UE population has a higher homogeneous utility, the UE population is more attractive to UEs.

According to the applied RA method on cache content placement and the greedy approach on UE cloud selection, it is highly possible that the cache mechanism does not work, and the cloud selection utilities drop down because of the congestions happened in the HEC, LEC, fronthaul or RF link in RRH *j*. This is because the cached contents in RRHs may be not interested by UEs, and UEs compete the computation and communication resources selfishly without cooperation in the greedy approach. Therefore, Algorithm 2 further implements the PE method co-working with CCPA (i.e., Algorithm 1) from step 16 to 30 to further realize rational cache content placement and UE cloud selections. As shown from step 16 to 30, if in a RRH *j*, the UE population has homogeneous utility small than average utility, for example ${\overset{ˇ}{U}}_{j}^{H}<{\bar{U}}_{j}^{H}$ in HUE population or ${\overset{ˇ}{U}}_{j}^{L}<{\bar{U}}_{j}^{L}$ in LUE population, that means the population is not in its optimal situation, and has issues like too many UEs in the population causing computation resource hungry, fronthaul capacity overfill, or most of the UEs in the population having weak RF link. In this case, the algorithm has to search the UEs in the population to find specific UEs to be moved to other population, which will potentially benefit the population. The UEs to be moved are the ones that have higher utility in other population than in current population. For example, if UE *i* in HUE population has $U_{i,j}^{L}>U_{i,j}^{H}$ (step 18), then UE *i* will be moved to LUE population (step 19). Another example is if UE *i* in LUE population has $U_{i,j}^{H}>U_{i,j}^{L}$ (step 21), then UE *i* will be moved to HUE population (step 22). After the UEs moving, the utilities of UEs selecting clouds and the average and homogeneous utilities of HUE population and LUE population will be updated (step 26–28). Most importantly, because the cloud selection of UEs changed, Algorithm 2 has to call Algorithm 1 to update $m_{jv}$ (∀v∈V) to get a more rational cache content placement in RRH *j* (step 25). The algorithm will finish, i.e., PE game coverages, when homogeneous utilities are higher than their average utilities both in HUE and LUE populations of all the RRHs in the set J, or the PE procedure running out of allowed step $S^{max}$. Then Algorithm 2 obtains the optimized cache content placement and cloud selection results: $\left(m_{jv},a_{ij}\right)$ ($\forall v\in V,\forall j\in J,\;\forall i\in R_{j}$).
**Algorithm 2:** Joint Solution on Cache Content Placement and User Task Offloading.
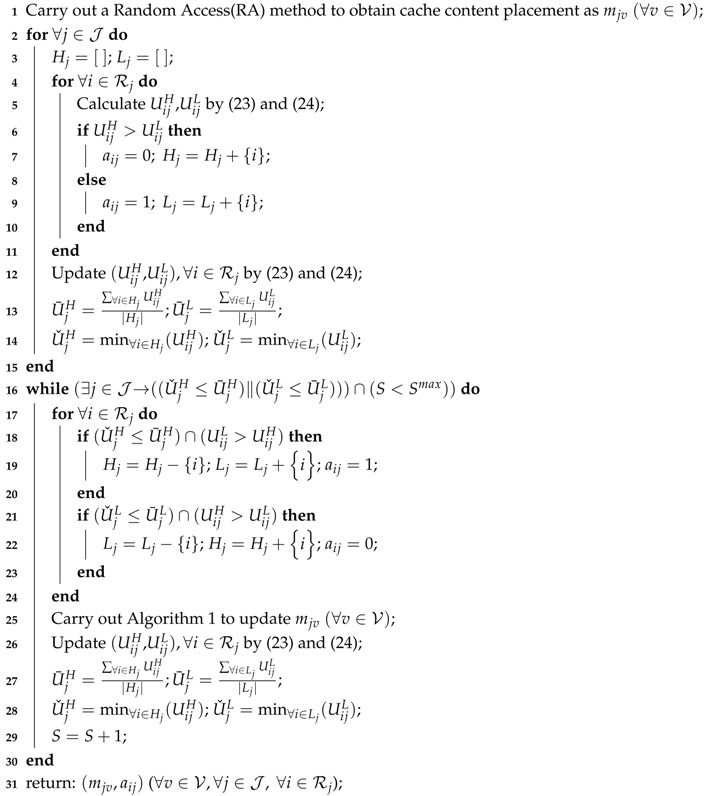


## 4. Simulation and Analysis

### 4.1. Simulation Outputs

**Figure 3:**
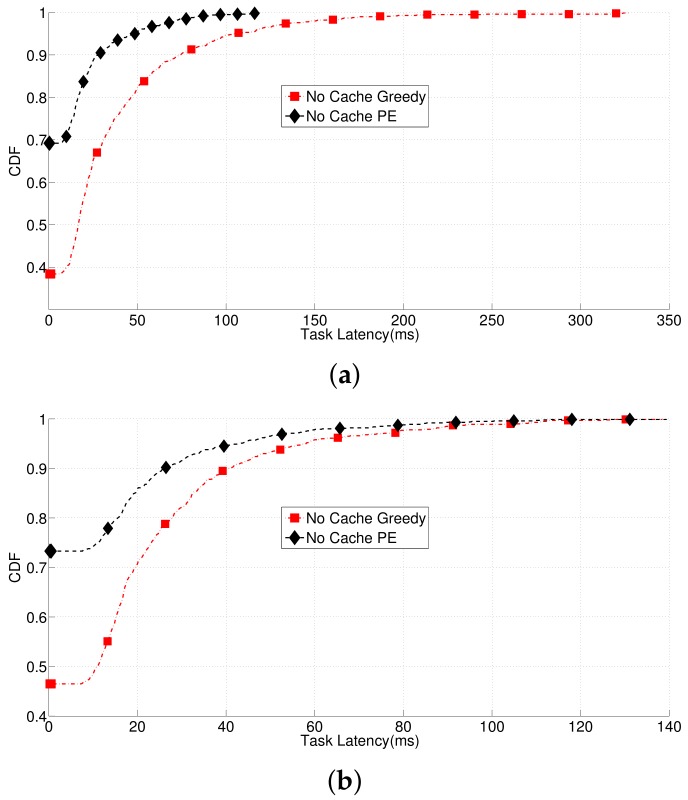
Comparisons of the UE latencies between the PE method and the Greedy method on user task offloading. (**a**): Data-process tasks; (**b**): Data-stream tasks.

This paper validates the GS-based CCPA algorithm and JCOS that jointly working with GS and PE, through a series of simulations using Matlab. The simulation parameters are listed in Table 2. In the simulation, we take user task latency as the main form of output to evaluate each solution, not particularly the energy cost. This is because the energy cost has already been considered by constraint C7 in problem P. In theory, as long as P being solved by proposed solutions under the constraints of C1−C8, CMM-CRAN can overall deliver low-latency and low-energy-cost services to mobile users. We first compare the latencies of all the UEs between the PE method and the Greedy method on user task offloading in Figure 3. During this comparison, we assume that the network does not have cache mechanism to better demonstrate the performance differences of the PE and Greedy methods on user task offloading. The Greedy method means a UE simply selects the cloud that offers itself the highest utility without cooperating with any other UEs, which has been discussed in Section 3. Obviously, this Greedy method will cause congestions in related clouds and fronthaul. Figure 3 shows the Cumulative Distribution Function (CDF) of all the UE latencies, and the PE method generally leads to lower UE latencies compared to the Greedy method. Especially when all the UE tasks are of the data-process, data-stream type, the average task latency led by the PE method are 14.30 ms and 14.49 ms respectively, and the average task latency led by the Greedy method are 45.29 ms and 29.06 ms respectively. We can get the same conclusion if working with the UE tasks of the multi-media or mixed type. However, because of the limited space of this paper, we will not show those results here.

**Figure 4:**
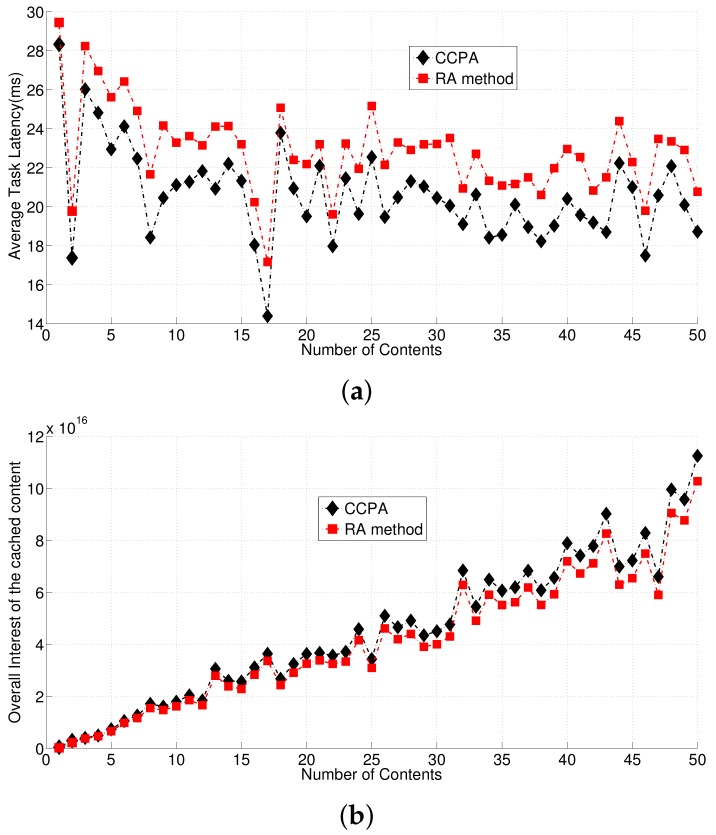
Comparisons of the average UE latencies and overall content interests led by CCPA and the RA method. (**a**): Average Task Latency; (**b**): Overall Content Interests.

Secondly, we compare the average latencies of the UEs between CCPA and the Random Access (RA) method on cache content placement in Figure 4. During this round of comparison, we assume the network employs the greedy-based cloud selection method to make sure CCPA and the RA method working on the same premise, where the network has congested cloud and fronthaul. The RA method means each content is randomly cached to RRHs, which has been discussed in Section 3. Figure 4 shows the average of the UE task latencies led by CCPA and RA method respectively, considering different number of contents. It is obvious that CCPA gives better performance on decreasing the UE task latency than the RA method. Especially when all the UE tasks are of the data-stream type, the average task latency led by CCPA is 20.60 ms, and the average task latency led by the RA method is 22.87 ms. Figure 4 also shows the overall interests of UEs to the cached content led by CCPA and the RA method respectively. It can be found that CCPA enables more interested content to be cached in RRHs, compared to the RA method. This validates that CCPA gives more benefits out of the cache mechanism to the network than the RA method.

**Figure 5:**
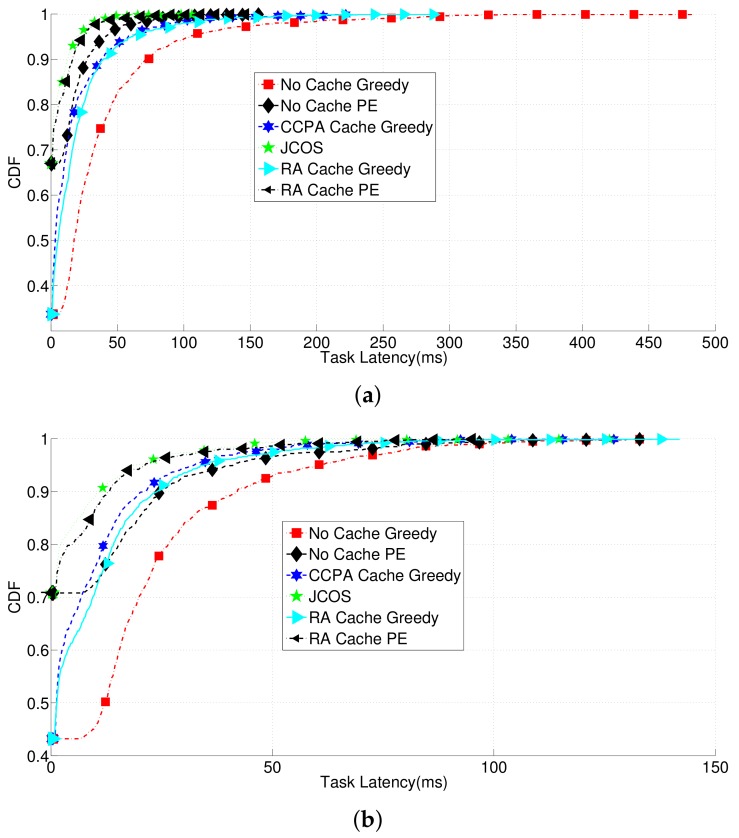
Comparisons of the UE latencies within different solutions on user task offloading and cache content placement. (**a**): Data-process tasks; (**b**): Data-stream tasks.

Finally, we give the overall performance comparisons considering all the possible cache content placements, user task offloading options, and JCOS. Figure 5 demonstrates the latency differences in CDF values led by varieties of combined solutions. Accordingly, the network that has no cache and employs the greedy method as the user task offloading solution gives the worst performance on UE task latency, while JCOS gives the best performance. However, the out-performance led by JCOS is marginal compared to the RA method working with the PE method. This is because the network is not quite congested after the working of the PE method, so the GS method cannot further obtain large amount of performance gain through cache content placement.

In general, these series of simulations validate that JCOS can improve the performance of CMM-CRAN by enormously decreasing the UE task latencies with limited energy cost on each user task. Therefore, JCOS is able to release the constraint of fronthaul in great extent to get the maximal performance gain of the network.

### 4.2. Analysis

In this section, we discuss the pros and cons of the GS and PE methods working in JCOS, with respect to their usabilities, scalabilities and computation complexities. In practice, JCOS with the GS and PE methods will be individually deployed in each RRH of CMM-CRAN. As RRH is in limited computation and storage capacities, JCOS should work lightly without causing too much resource cost, and be easily deployed to any other type of cache and multi-layer MEC enabled C-RAN. Therefore, JCOS has to have high usability, scalability and low computation complexity.

First, CCPA provides an effective solution to the cache content placement problem of this paper. CCPA takes matching players’ (RRHs or contents) preferences over the opposite players into account to reach a many-to-many stable matching. With the stable matching, each RRH then has the most interested contents cached in its storage to save data transmissions in fronthaul. This has been validated in simulation that CCPA works better than the RA method. In practice, CCPA collects network parameters from CMM-CRAN, including the fronthaul capacity, RF link capacity, RRH cloud capacity, RRHs’ interests to contents and the task offloading situations, to calculate the players’ preferences. Unfortunately, these network parameters may not always available to CCPA, and the parameter collections cost information exchange overhead within UEs, RRHs and SPSs. The parameters defect and collection overhead will affect the performance of CCPA. Considering scalability and computational complexity, the time complexity of CCPA is not high, which is $O(n^{2})$ where *n* is the total number of RRHs and contents. The scalability of CCPA is limited by the computation capacity of each RRH and the collecting of numbers of parameters for the matching. Especially, if the CMM-CRAN has enormous amount of contents to be cached into each RRHs, CCPA may take long time to coverage in RRH. With this limited scalability, when social-aware content updates quickly in CMM-CRAN, CCPA may not be in practical usage. To alleviate the problem, LEC deployed in RRH can help the computing of CCPA. Also the CCPA needs further improvement to decrease its computation complexity.

Second, the PE method used in JCOS is a classic evolutionary game to solve the user task offloading problem of this paper. According to Algorithm 2, the main activities of the PE method in JCOS are to carry out heuristic searching to make sure each UE population has member UEs with relatively high cloud selection utilities, while iteratively working with CCPA. The PE method works out the user task offloading problem using an evolutionary game with a series of utility comparisons. It involves less computation and training time, but gives a better result than the greedy method, which has been validated by simulations in this section. However, there are two challenges concerning the PE method working in JCOS. First, there is a possibility that the heuristic search cannot find the optimal solution, but only find a local optimization. This is because the heuristic searching is for each UE, out of the UEs’ cloud selection population, without looking at the global problem P. Even through the PE game itself tries to reach a global equilibrium to benefit all of the UEs, this issue still exists. Second, the heuristic search may cause the UEs’ population regrouping to fall into a backwards versus forwards dilemma, where a UE may be regrouped back to its previous UE population without reaching an optimization. Those two challenges will escalate if the UE group I is large, leading to Algorithm 2 not being able to converge after a long time of running. The time complexity of Algorithm 2 is $O(n^{4})$, where *n* is the size of the UEs under consideration. As the number of UEs in a RRH is relatively low, this makes JCOS scalable and suitable to work in CMM-CRAN, when the algorithm working in a distributed way in each RRH.

## 5. Conclusions

This paper provides a Joint Cache content placement and user task Offloading Solution (JCOS) to a cache and multi-layer MEC enabled C-RAN. The CCPA in JCOS is to make sure the UE most interested social-aware contents being cached into the storage-constrained RRHs to further save fronthaul data transmission. The PE method working in JCOS with CCPA provides a game theory-based cloud selection strategy to realized the user task offloading of all the UEs. JCOS has been well validated in this paper, but they still need improvements in terms of usability and scalability.

In future work, we will consider cache content placement in a distributed way under the constraints of limited network parameters. We intend to have better cache solution with improved scalability. In addition, the PE method and CCPA currently work in JCOS in a loose-coupled way, and mainly obtain outputs through heuristic searching with no optimal result guaranteed. We need to design a more effective joint solution. Specifically, we will explore the possibility that the PE method converges to global optimization with limited computation overhead for the user task offloading problem. Meanwhile, we will looking at other alternative solutions out of matching theory and machine learning methodology to give better cache and UE task offloading strategies. In addition, the energy cost of CMM-CRAN is not directly considered in JCOS in this paper. To make CMM-CRAN be a greener radio network, we should re-formulate the energy cost in the joint solution and improve the cache and user task offloading results in future work. 

## Figures and Tables

**Table 1:** UEs’ sensitivenesses to cloud selection related elements.

Elements	UEs’ Sensitivenesses to Each Element			
	Voice	Data-Process	Stream	Multi-Media
Cloud Capacity	2(low)	8(high)	5(medium)	9(high)
Fh Constraint	8(high)	1(low)	6(medium)	8(high)
RF Constraint	9(high)	3(low)	4(medium)	7(high)

**Table 2:** Parameters of the Simulation.

Parameter	Value
Number of RRH: *J*	20
Number of UEs in a RRH: $|R_{j}|$	10∼50
Number of contents: *V*	20∼200
Capacity of a LEC: $f_{j,max}^{L}$	$|R_{j}\left|\times (5∼10\right)$
Capacity of HEC: $f_{max}^{H}$	$|R_{j}\left|\times J\times (10∼20\right)$
CPU requirement of voice task	1∼5
CPU requirement of data process task	30 ∼ 50
CPU requirement of data stream task	5∼20
CPU requirement of multi-media task	20∼40
Sensitivity for sensitized element	7∼9
Sensitivity for medium-sensitized element	4∼6
Sensitivity for non-sensitized element	1∼3
Cache capacity of each RRH	500Mb∼2000Mb
Size of a content	5Mb∼20Mb
Data rate of Fronthaul per RRH	100 Mbs ∼ 200 Mbs
Data rate of a RF link	1Mbs∼10Mbs
The interest of a UE to a content: $t_{iv}$	10∼100
Maximal allowed task latency: $T_{i,max}$	200 ms
Maximal allowed energy cost of each user task: $E_{i,max}$	5 J
Maximal step of the PE procedure: $S^{max}$	100

## Abbreviations

The following abbreviations are used in this manuscript:

Cloud Radio Access Network	C-RAN
Cache and Multi-layer MEC enabled C-RAN	CMM-CRAN
Remote Radio Head	RRH
Proportional Fairness	PF
Population Evolution	PE
User Equipment	UE
Fog computing-based RAN	F-RAN
High-level Edge Cloud	HEC
Cache Content Placement Algorithm	CCPA
Radio Block	RB
Multi-Dimension Multiple-Choice Knapsack	MMCK
Cumulative Distribution Function	CDF
Mobile Edge Computing	MEC
Service Provide Server	SPS
Joint Cache content placement and task Offloading Solution	JCOS
Gale-Shaply	GS
Analytic Hierarchy Process	AHP
Base Band Unit	BBU
Maximum Distance Separable	MDS
Low-level Edge Cloud	LEC
Signal to Interference plus Noise Ratio	SINR
Computation Block	CB
Random Access	RA
Orthogonal Frequency Division Multiplexing	OFDM
